# Admission Hydration Status and Ischemic Stroke Outcome—Experience from a National Registry of Hospitalized Stroke Patients

**DOI:** 10.3390/jcm10153292

**Published:** 2021-07-26

**Authors:** Yoav Eizenberg, Ehud Grossman, David Tanne, Silvia Koton

**Affiliations:** 1 Department of Endocrinology, Clalit Health Services, Sackler Faculty of Medicine, Tel Aviv University, Tel Aviv-Yaffo District, Tel Aviv 6997801, Israel; yoave1@yahoo.com; 2 Internal Medicine Wing, The Chaim Sheba Medical Center, Sackler Faculty of Medicine, Tel Aviv University, Tel Hashomer, Tel Aviv 6997801, Israel; ehud.grossman@sheba.health.gov.il; 3 Rambam Health Care Campus, Stroke and Cognition Institute, Haifa 3109601, Israel; d_tanne@rambam.health.gov.il; 4 Stanley Steyer School of Health Professions, Sackler Faculty of Medicine, Tel-Aviv University, Tel Aviv 6997801, Israel

**Keywords:** stroke, dehydration, outcomes, complications, disability, death, creatinine

## Abstract

Reduced intravascular volume upon ischemic stroke (IS) admission has been associated with in-hospital complications, disability, and reduced survival. We aimed to evaluate the association of the urea-to-creatinine ratio (UCR) with disability or death at discharge, length of stay, in-hospital complications, and mortality during the first year. Using a national registry, we identified hospitalized IS patients without renal failure. Disability or death at discharge, length of stay, in-hospital complications, and mortality during the first year were studied by UCR, and associations between UCR levels and each outcome were assessed adjusting for age, sex, stroke severity, comorbidities, use of statins, and use of diuretics. In total, 2212 patients were included. Levels (median (25–75%)) for the main study variables were: urea 5.16 (3.66–6.83) mmol/L; creatinine 80 (64–92) µmol/L; and UCR 65 (58–74). Levels of UCR were significantly higher in patients with disability or death at discharge (*p* < 0.0001), those with complications during hospitalization (*p* = 0.03), those with infection during hospitalization (*p* = 0.0003), and those dead at 1 year (*p* < 0.0001). Analysis by UCR quartile showed that rates of disability or death at discharge, infections, complications overall, and death at 1 year in patients with UCR in the 4th quartile were significantly higher than in others. Risk-factor-adjusted analysis by UCR quartiles demonstrated an inconsistent independent association between UCR and disability or death after ischemic stroke. A high 1-year mortality rate was observed in IS patients with elevated UCR, yet this finding was not statistically significant after controlling for risk factors. Our study shows inconsistent associations between hydration status and poor functional status at discharge, and no association with length of stay, in-hospital complications (infectious and overall), and 1-year mortality.

## 1. Introduction

Reduced intravascular volume, commonly referred to as dehydration, is a common finding upon admission of patients with ischemic stroke (IS), reported in 29–70% of patients [1]. It has been associated with in-hospital complications [2,3,4,5,6,7,8,9], poor functional outcome at discharge [4,10,11], poor functional outcome at 30 days [12], and reduced survival at 90 days [13,14]. Current guidelines for treatment of IS recommend maintaining euvolemia [15], but accurately determining the intravascular status in IS patients remains a difficult task. Several methods to assess intravascular volume have been suggested; however, the blood urea nitrogen (BUN)/creatinine ratio and the urea/creatinine ratio (UCR) continue to be the most commonly used and widely accepted [1].

Using data from the National Acute Stroke Israeli Survey (NASIS) registry of hospitalized stroke patients, we aimed to evaluate the association of hydration status in IS, as measured by UCR, with stroke severity on admission, in-hospital complications, and mortality during the first year after ischemic stroke.

## 2. Materials and Methods

### 2.1. Study Design and Setting

The NASIS prospective hospital-based national registry was prospectively performed during 2-month periods triennially [16,17] (February–March 2004, March–April 2007, April–May 2010, April–May 2013, and April–May 2016). All consecutive patients hospitalized due to acute stroke or transient ischemic attack age 18 and over nationwide were included. The NASIS registry was approved by the ethical committees of the participating medical centers.

Data on creatinine and urea levels were collected in NASIS 2013 and 2016. In total, 4617 patients were included in these two periods, among them 3171 patients with ischemic stroke. Complete data on creatinine and urea levels were available for 2767/3171 (87.3%). No significant differences between patients with and without data on urea and creatinine levels were observed in the distribution of demographics (age (*p* = 0.52), sex (*p* = 0.73)), or in risk factors and clinical characteristics including hypertension (*p* = 0.99), diabetes (*p* = 0.77), current smoking (*p* = 0.89), prior stroke (*p* = 0.40), prior heart disease (*p* = 0.62), use of statins (*p* = 0.29), and use of diuretics (*p* = 0.19). We excluded 555 participants with renal failure (by history or with creatinine >1.5 mg/dL). Therefore, the final sample for the present analysis included 2212 ischemic stroke patients.

### 2.2. Data Collection

Coordinating physicians prospectively collected data from all the departments of the participating medical centers. A structured data form including demographics, risk factors and co-morbidities, stroke characteristics, in-hospital management, and outcome was used for collecting the information. Urea and creatinine levels were measured at local labs of all medical centers on admission. Mortality was prospectively assessed at 1 year after stroke by means of matching patients’ files with national mortality data.

### 2.3. Definition of Main Variables

Urea/creatinine ratio (UCR) was used to evaluate level of hydration on admission. Severity of stroke on admission was categorized into five NIH Stroke Scale (NIHSS) [18] levels, as defined by the NASIS protocol. Severe stroke was defined as NIHSS ≥ 16. Functional outcome was evaluated at discharge according to the modified Rankin Scale (mRS) [19] and categorized as mRS 0–1; 2–3; 4–5; and 6 (death). Based on the mRS category, we defined disability at discharge or death as mRS ≥ 2 [20]. Complications during hospitalization were defined as neurological, cardiac, infectious, or bleeding during hospitalization. Infection included pneumonia, urinary tract infection, sepsis, fever, and other infection during hospitalization. Complications during hospitalization were reported at discharge and were analyzed overall and by studying infections as a separate outcome. We also assessed length of stay as a continuous variable and defined prolonged hospitalization as ≥7 days.

### 2.4. Statistical Analysis

Baseline characteristics, stroke severity on admission, and complications during hospitalization were studied by UCR quartiles, as well as by looking at UCR level as a continuous variable. UCR shows a distribution skewed to the right with a mean of 38.6 and a median of 36.7. Therefore, associations between UCR level and all complications and infections during hospitalization were studied with the Wilcoxon test, while the Spearman correlation test was used to assess association between UCR level and length of stay.

In the multivariate analysis, logistic regression models were used to study associations between UCR levels as a continuous variable as well as UCR quartiles and each of the outcome variables, controlling for potential confounders. Odds ratio (OR) and 95% confidence interval (95% CI) associated with 1-SD UCR for each outcome were presented, adjusted for age and sex (Model 1) and further accounting for the following risk factors, selected a priori, based on clinical discretion and recently published evidence [21]: stroke severity, hypertension, diabetes mellitus, smoking, history of atrial fibrillation, prior stroke, prior heart disease, peripheral artery disease, disability before the stroke (mRS ≥ 2 before the stroke), use of statins, and use of diuretics for 1 week or more before symptoms onset, in addition to age and sex (Model 2). Risk estimates by UCR quartiles using the lowest UCR quartile as reference group were computed with logistic regression models adjusting for risk factors as described (Model 1 and Model 2).

In a sensitivity analysis, potential associations between UCR and the outcome variables were evaluated in 236/2212 (10.7%) patients undergoing reperfusion therapy (either intravenous thrombolysis or mechanical thrombectomy).

Analyses were performed with SAS 9.4 (SAS, SAS Institute, Cary, NC, USA).

## 3. Results

### 3.1. Participants’ Characteristics

In total, 2212 ischemic stroke patients were included, with 47.6% being women. Mean (SD) age was 70.3 (13.2) years. Median levels (25–75%) for the main study variables were as follows: urea 5.16 (3.66–6.83) mmol/L (31 (22–41) mg/dL); creatinine 80 (64–92) µmol/L (0.90 (0.72–1.04) mg/dL); UCR 65 (57–74) (36.69 (25.00–47.77)); and length of stay 5 (3–9) days. Severe stroke was reported for 8.4% of patients, while 65.6% were diagnosed with minor stroke (NIHSS ≤ 5). Complications during hospitalization were observed in 403 (18.2%) participants, while 242 (11.0%) participants were diagnosed with infections and 260 (11.8%) died during the first year after IS.

Characteristics of patients by UCR quartile are shown in Table 1. In general, rates of cardiovascular risk factors, except for smoking at time of stroke, were higher in participants in the highest UCR quartile compared with lower quartiles. Statistically significant differences in baseline characteristics by UCR quartiles were observed for demographics and most clinical factors, as shown in Table 1.

### 3.2. Associations Between UCR and Poor Outcome after Stroke

Levels of UCR were significantly higher in patients with complications during hospitalization (*p* = 0.03), those with infection during hospitalization (*p* = 0.0003), those with disability or death at discharge (*p* < 0.0001), and those dead at 1 year (*p* < 0.0001). No correlation was observed between UCR and length of stay (r = 0.01, *p* = 0.56) or prolonged hospitalization (≥7 days, *p* = 0.46).

Analysis by UCR quartile showed that the rate of death at 1 year in patients with UCR in the 4th quartile was higher than in others (Table 2). Statistically significant differences in rates of outcome variables by UCR quartiles were observed for disability, infection during hospitalization, and 1-year mortality (Table 2).

Our findings suggest non-linear associations between UCR levels and length of stay and complications during hospitalization (Table 2).

Results of the multivariate analysis of associations between level of UCR and the studied outcomes show that following adjustment for age, sex, stroke severity, and cardiovascular risk factors (Model 2), no significant associations were observed between UCR as a continuous variable and stroke outcome (Table 3).

In the analysis by UCR quartiles, age- and sex-adjusted estimates (ORs, 95% CIs) for disability or death at discharge were increased for patients with UCR in the 2nd, 3rd, and 4th quartiles, compared with those in the first quartile. These findings were consistent after controlling for risk factors (Model 2) for patients in the 2nd quartile of UCR (Table 4). Unadjusted estimates (ORs, 95% CIs) for in-hospital complications, infections, and 1-year mortality were increased for patients in the 4th compared with the 1st UCR quartile (Table 4). However, these findings were not consistent following adjustment (Table 4, Model 2). Interestingly, a negative association between complications and UCR was observed for patients in the 3rd quartile compared with the 1st quartile (adjusted OR 0.66, 95% CI 0.45–0.97) (Table 4).

In the sensitivity analysis of data on patients undergoing reperfusion therapy during hospitalization, no significant associations were observed between UCR level and the study outcomes in the unadjusted models or in models adjusted for age and sex (Model 1). We present the results of this analysis in Table 5. Due to the small number of patients with reperfusion therapy, further adjustment was not conducted in the regression models.

## 4. Discussion

Dehydration is common in IS patients and has been associated with several unfavorable outcomes. Yet, our study shows inconsistent association between hydration status and poor functional status at discharge and no association with length of stay, in-hospital complications (infectious and overall), and 1-year mortality.

Dehydration, characterized by an elevated UCR, has been linked to disability or death at discharge in several studies to this point. Compatible with our study, Rowat et al. showed that a UCR >80 (UCR > 45) was associated with dependency or death at hospital discharge [10]. However, in our study, the risk for a poor functional outcome was significant at a lower UCR, with loss of significance in higher UCR quartiles. In another study, IS patients with a BUN/creatinine ratio >26 (BUN/Creatinine ratio > 15) (equivalent to a UCR > 56 (UCR ≥ 32)) had lower mRS scores even after controlling for age, sex, hypertension, diabetes, admission NIHSS score, and infection, but no other UCR cutoffs were explored [4]. It is noteworthy that the sensitivity analysis including only patients with reperfusion therapy did not show associations between UCR and any of the study outcomes, as was also demonstrated by Wu et al. with a larger sample [11].

Our results do not support the association of UCR with length of stay. Smithard et al. investigated the relationship of dysphagia, a common pathophysiological cause of dehydration in IS, with length of stay and also did not found an association [22]. A more recent interventional study showed a shorter length of stay in IS patients given intravenous saline based on a BUN/creatinine ratio > 26 (BUN/creatinine ratio > 15) (equivalent to a UCR > 56 (UCR ≥ 32)) as a marker of dehydration [23]. This study also reported a reduced infection rate in patients receiving intravenous saline based on the same BUN/creatinine ratio cutoff. Moreover, in a large cohort study by Liu et al., a higher in-hospital infection rate was observed in IS patients with an elevated BUN/creatinine ratio [4]. However, our data reflect a stable infection rate over a wide range of UCRs. This may be due to differences in studies’ populations or in local acute care procedures. Our data also show that overall in-hospital complications are not associated with hydration status.

After adjusting for several potential confounders, we found that dehydration was not associated with increased mortality in the first year after the stroke admission. On the contrary, Bhalla et al. found that increased plasma osmolality is significantly associated with mortality at 90 days, although with borderline significance [13], possibly attributed to a small number of patients. Moreover, data adapted from a large prospective trial showed that mortality at 90 days was associated with an elevated urea level but not with an elevated UCR [14], perhaps due to concomitant underlying renal dysfunction [24]. To the best of our knowledge, previous studies have not recorded mortality data for longer periods.

Our study has several strengths. First, it uses a national registry of considerable size including all hospitalized stroke patients in the relevant time periods. Second, hospitalization data were collected by well-trained experienced teams, minimizing data mishandling. Third, stroke severity on admission and functional status were assessed using validated quantitative measures (NIHSS and mRS, respectively). However, the present study has some limitations. NASIS is a hospital-based registry; therefore, it is possible that minor ischemic strokes not referred to the hospital were not recorded, although hospitalization of all stroke cases is the current policy in Israel. Hydration status was pragmatically defined by UCR, not by more reliable measures such as plasma osmolarity. In addition, data on pre-admission factors (e.g., time to admission) and post-admission factors (e.g., varying hydration protocols among participating hospitals) that may affect hydration status were only partially available in NASIS. Although NASIS includes broad patient-level information, data on NIHSS score as a continuous variable as well as severity or duration of baseline medical conditions were not available. Lastly, residual confounding is a possibility as in any observational study.

## 5. Conclusions

Our study shows inconsistent associations between hydration status and poor functional status at discharge and no association with length of stay, in-hospital complications (infectious and overall), and 1-year mortality. As current literature continues to be inconclusive on this matter, large prospective studies are needed to reach more firm conclusions.

## Figures and Tables

**Table 1:** Baseline characteristics of ischemic stroke patients by urea/creatinine ratio (UCR) quartiles.

	UCR Quartiles				*p* for Trend
	<57 *n* = 559	57–65 *n* = 547	65–74 *n* = 553	>74 *n* = 553	
Age (SD), years	68.10 (13.15)	67.43 (13.23)	71.14 (12.79)	74.33 (12.48)	<0.0001
Women	229 (41.0)	213 (38.9)	245 (44.3)	366 (66.2)	<0.0001
Stroke severity by NIHSS					<0.0001
≤5	399 (71.4)	373 (68.2)	363 (65.6)	315 (57.0)	
6–10	100 (17.9)	98 (17.9)	116 (21.0)	120 (21.7)	
11–15	25 (4.5)	37 (6.8)	39 (7.1)	41 (7.4)	
16–20	22 (3.9)	24 (4.4)	20 (3.6)	55 (10.0)	
>20	13 (2.3)	15 (2.7)	15 (2.7)	22 (4.0)	
Severe stroke (NIHSS ≥ 16)	35 (6.3)	39 (7.1)	35 (6.3)	77 (13.9)	<0.0001
Hypertension	414 (74.3)	401 (73.7)	439 (79.8)	450 (81.8)	0.0003
Diabetes	244 (43.7)	218 (39.9)	231 (41.9)	245 (44.5)	0.66
Current smoking	144 (26.1)	144 (26.8)	128 (23.6)	95 (17.4)	0.0003
Prior stroke	114 (20.6)	150 (27.6)	155 (28.2)	153 (28.5)	0.0034
Atrial fibrillation	67 (12.1)	98 (18.0)	112 (20.5)	155 (28.1)	<0.0001
Prior heart disease	136 (24.3)	132 (24.1)	146 (26.4)	145 (26.2)	0.34
Peripheral arterial disease	20 (3.6)	23 (4.2)	24 (4.4)	39 (7.1)	0.009
Disability before the stroke	116 (21.3)	129 (24.4)	165 (30.7)	220 (41.6)	<0.0001
Statin medication use	274 (50.9)	261 (50.3)	261 (50.4)	272 (51.6)	0.83
Diuretics use	97 (17.93)	111 (22.75)	96 (19.43)	144 (28.29)	0.0006

Figures represent number and percentage if not otherwise specified. Prior heart disease was defined as chronic heart failure, prior myocardial infarction, atrial fibrillation, or valve disease. Disability before the stroke was defined as mRS ≥ 2 before stroke. Use of statin medication and diuretics was defined as treatment for at least 7 days before onset of stroke symptoms.

**Table 2:** Functional status, length of stay, complications during hospitalization, and 1-year mortality in ischemic stroke patients by urea/creatinine ratio (UCR) level.

	UCR QUARTILES				*p* for Trend
	<57 *n* = 559	57–65 *n* = 547	65–74 *n* = 553	>74 *n* = 553	
Modified Rankin Scale (mRS)					<0.0001
0–1	285 (51.08)	241 (44.14)	222 (40.14)	182 (32.91)	
2–3	168 (30.11)	191 (34.98)	201 (36.35)	163 (29.48)	
4–5	85 (15.23)	95 (17.40)	115 (20.80)	176 (31.83)	
6	20 (3.58)	19 (3.48)	15 (2.71)	32 (5.79)	
Disability or death (discharge mRS ≥ 2)	273 (48.92)	305 (55.86)	331 (59.86)	371 (67.90)	<0.0001
Length of stay, mean (SD), days	8.42 (18.74)	7.80 (8.51)	7.47 (9.10)	9.24 (18.52)	0.19
Complications during hospitalization	99 (17.74)	92 (16.85)	86 (15.55)	126 (22.83)	0.06
Infection during hospitalization	53 (9.50)	48 (8.79)	55 (9.95)	86 (15.61)	0.0011
1-year mortality	61 (12.45)	50 (10.99)	58 (13.52)	91 (20.97)	0.0002

Figures represent number and percentage if not otherwise specified.

**Table 3:** Stroke outcome estimates (ORs (95% Cis)) associated with level of urea/creatinine ratio (UCR).

	OR (95% CI) for 1-SD in UCR		
	Unadjusted	Model 1	Model 2
Disability or death (discharge mRS ≥ 2)	**1.38 (1.23–1.55)**	**1.18 (1.06–1.31)**	1.07 (0.97–1.19)
Length of stay ≥7 days	1.01 (0.93–1.10)	0.98 (0.90–1.07)	0.95 (0.85–1.05)
Complications during hospitalization	1.07 (0.97–1.18)	0.99 (0.88–1.11)	0.85 (0.72–1.01)
Infection during hospitalization	**1.14 (1.03–1.27)**	1.07 (0.95–1.21)	0.95 (0.78–1.17)
1-year mortality	**1.19 (1.06–1.34)**	1.11 (0.97–1.27)	0.99 (0.81–1.21)

Model 1 adjusted for age and sex. Model 2 adjusted for age, sex, stroke severity, hypertension, diabetes, current smoking, atrial fibrillation, prior stroke, prior heart disease, peripheral artery disease, disability before the stroke, use of statins, and use of diuretics for1 week or more before symptoms onset. Statistically significant estimates are shown in bold.

**Table 4:** Stroke outcome estimates (ORs (95% Cis)), by quartiles of urea/creatinine ratio (UCR).

	OR (95% CI)			
	UCR	Unadjusted	Model 1	Model 2
Disability or death (discharge mRS ≥ 2)	1st quartile	1 (Reference)	1 (Reference)	1 (Reference)
	2nd quartile	**1.32 (1.04–1.67)**	**1.44 (1.12–1.85)**	**1.38 (1.01–1.91)**
	3rd quartile	**1.56 (1.23–1.97)**	**1.38 (1.07–1.77)**	1.21 (0.87–1.67)
	4th quartile	**2.13 (1.67–2.71)**	**1.59 (1.22–2.06)**	1.14 (0.80–1.63)
Length of stay ≥ 7 days	1st quartile	1 (Reference)	1 (Reference)	1 (Reference)
	2nd quartile	0.94 (0.69–1.28)	0.99 (0.78–1.26)	0.92 (0.70–1.20)
	3rd quartile	0.85 (0.62–1.17)	0.83 (0.65–1.06)	0.62 (0.58–1.02)
	4th quartile	1.37 (1.02–1.84)	0.94 (0.74–1.21)	0.77 (0.58–1.02)
Complications during hospitalization	1st quartile	1 (Reference)	1 (Reference)	1 (Reference)
	2nd quartile	0.94 (0.69–1.28)	0.97 (0.70–1.33)	0.82 (0.56–1.19)
	3rd quartile	0.84 (0.61–1.15)	0.75 (0.54–1.03)	**0.66 (0.45–0.97)**
	4th quartile	**1.37 (1.02–1.84)**	1.04 (0.76–1.41)	0.76 (0.53–1.11)
Infection during hospitalization	1st quartile	1 (Reference)	1 (Reference)	1 (Reference)
	2nd quartile	0.92 (0.61–1.38)	0.95 (0.62–1.44)	0.74 (0.44–1.23)
	3rd quartile	1.05 (0.71–1.57)	0.91 (0.60–1.39)	0.78 (0.47–1.28
	4th quartile	**1.76 (1.22–2.54)**	1.26 (0.86–1.85)	0.91 (0.57–1.45)
1-year mortality	1st quartile	1 (Reference)	1 (Reference)	1 (Reference)
	2nd quartile	0.87 (0.58–1.29)	0.89 (0.59–1.35)	0.72 (0.43–1.21)
	3rd quartile	1.10 (0.75–1.62)	0.88 (0.59–1.32)	0.83 (0.51–1.35)
	4th quartile	**1.87 (1.31–2.66)**	1.29 (0.88–1.88)	0.97 (0.61–1.55)

Model 1 adjusted for age and sex. Model 2 adjusted for age, sex, stroke severity, hypertension, diabetes, current smoking, atrial fibrillation, prior stroke, prior heart disease, peripheral artery disease, disability before the stroke, use of statins, and use of diuretics for1 week or more before symptoms onset. Statistically significant estimates are shown in bold.

**Table 5:** Stroke outcome estimates (ORs (95% Cis)) associated with level of urea/creatinine ratio (UCR) in patients treated with reperfusion therapy, *n* = 236.

	OR (95% CI) for 1-SD in UCR	
	Unadjusted	Model 1
Disability or death (discharge mRS ≥ 2)	0.85 (0.59–1.23)	0.74 (0.50–1.11)
Length of stay ≥ 7 days	0.99 (0.69–1.42)	0.90 (0.62–1.32)
Complications during hospitalization	1.24 (0.85–1.82)	1.14 (0.76–1.71)
Infection during hospitalization	1.39 (0.91–2.14)	1.34 (0.85–2.09)
1-year mortality	0.97 (0.56–1.71)	0.84 (0.45–1.54)

Model 1 adjusted for age and sex.

## Data Availability

The data presented in this study are available on request from the corresponding author. The data are not publicly available due to privacy restrictions.

## References

[1] Bahouth M.N., Gottesman R.F., Szanton S.L. (2018). Primary ‘dehydration’ and acute stroke: A systematic research review. J. Neurol..

[2] Lin L.C., Yang J., Weng H.-H., Hsiao C., Lai S.L., Fann W. (2011). Predictors of early clinical deterioration after acute ischemic stroke. Am. J. Emerg. Med..

[3] Lin L., Fann W., Chou M., Chen H., Su Y., Chen J. (2011). Urine specific gravity as a predictor of early neurological deterioration in acute ischemic stroke. Med. Hypotheses.

[4] Liu C.-H., Lin S.-C., Lin J.-R., Yang J.-T., Chang Y.-J., Chang C.-H., Chang T.-Y., Huang K.-L., Ryu S.-J., Lee T.-H. (2014). Dehydration is an independent predictor of discharge outcome and admission cost in acute ischaemic stroke. Eur. J. Neurol..

[5] Mohanty S., Tripathi B.K., Bhatia K., Gupta B., Mittal M.K. (2015). Predictors of early neurological deterioration in patients with acute ischaemic stroke with special reference to blood urea nitrogen (BUN)/creatinine ratio & urine specific gravity. Indian J. Med. Res..

[6] Bahouth M.N., Bahrainwala Z., Hillis A.E., Gottesman R.F. (2016). Dehydration Status is Associated with More Severe Hemispatial Neglect After Stroke. Neurologist.

[7] Li S.-S., Yin M.-M., Zhou Z.-H., Chen H.-S. (2017). Dehydration is a strong predictor of long-term prognosis of thrombolysed patients with acute ischemic stroke. Brain Behav..

[8] Deng L., Qiu S., Wang C., Bian H., Wang L., Li Y., Wu B., Liu M. (2019). Effects of the blood urea nitrogen to creatinine ratio on haemorrhagic transformation in AIS patients with diabetes mellitus. BMC Neurol..

[9] Shi Z., Zheng W.C., Yang H., Fu X.L., Cheng W.Y., Yuan W.J. (2019). Contribution of dehydration to END in acute ischemic stroke not mediated via coagulation activation. Brain Behav..

[10] Rowat A., Graham C., Dennis M. (2012). Dehydration in Hospital-Admitted Stroke Patients. Stroke.

[11] Wu F.-F., Hung Y.-C., Tsai Y.H., Yang J.-T., Lee T.-H., Liow C.-W., Lee J.-D., Lin C.-J., Peng T.-I., Lin L.-C. (2017). The influence of dehydration on the prognosis of acute ischemic stroke for patients treated with tissue plasminogen activator. BMC Cardiovasc. Disord..

[12] Schrock J.W., Glasenapp M., Drogell K. (2012). Elevated blood urea nitrogen/creatinine ratio is associated with poor outcome in patients with ischemic stroke. Clin. Neurol. Neurosurg..

[13] Bhalla A., Sankaralingam S., Dundas R., Swaminathan R., Wolfe C.D.A., Rudd A.G. (2000). Influence of raised plasma osmolality on clinical outcome after acute stroke. Stroke.

[14] Billington C.K., Appleton J.P., Berge E., Sprigg N., Glover M., Bath P.M.W. (2018). Impact of hydration status on haemodynamics, effects of acute blood pressure-lowering treatment, and prognosis after stroke. Br. J. Clin. Pharmacol..

[15] Powers W.J., Rabinstein A.A., Ackerson T., Adeoye O.M., Bambakidis N.C., Becker K., Biller J., Brown M., Demaerschalk B.M., Hoh B. (2018). 2018 Guidelines for the Early Management of Patients with Acute Ischemic Stroke: A Guideline for Healthcare Professionals From the American Heart Association/American Stroke Association. Stroke.

[16] Tanne D., Goldbourt U., Koton S., Grossman E., Koren-Morag N., Green M.S., Bornstein N.M. (2006). National Acute Stroke Israeli Survey Group a National Survey of Acute Cerebrovascular Disease in Israel: Burden, Management, Outcome and Adherence to Guidelines. Isr. Med. Assoc. J..

[17] Koton S., Tanne D., Green M.S., Bornstein N.M. (2010). Mortality and Predictors of Death 1 Month and 3 Years after First-Ever Ischemic Stroke: Data from the First National Acute Stroke Israeli Survey (NASIS 2004). Neuroepidemiology.

[18] National Institute of Neurological Disorders and Stroke rt-PA Stroke Study Group (1995). Tissue Plasminogen Activator for Acute Ischemic Stroke. N. Engl. J. Med..

[19] Herndon R.M. (1997). Handbook of Neurologic Rating Scales.

[20] Koton S., Telman G., Kimiagar I., Tanne D. (2013). Gender differences in characteristics, management and outcome at discharge and three months after stroke in a national acute stroke registry. Int. J. Cardiol..

[21] Torres-Aguila N.P., Carrera C., Muiño E., Cullell N., Carcel-Marquez J., Gallego-Fabrega C., González-Sánchez J., Bustamante A., Delgado P., Ibañez L. (2019). Clinical Variables and Genetic Risk Factors Associated with the Acute Outcome of Ischemic Stroke: A Systematic Review. J. Stroke.

[22] Smithard D., O’Neill P., Park C., Morris J., Wyatt R., England R., Martin D. (1996). Complications and Outcome After Acute Stroke. Stroke.

[23] Lin W.-C., Shih H.-M., Lin L.-C. (2015). Preliminary Prospective Study to Assess the Effect of Early Blood Urea Nitrogen/Creatinine Ratio-Based Hydration Therapy on Poststroke Infection Rate and Length of Stay in Acute Ischemic Stroke. J. Stroke Cerebrovasc. Dis..

[24] Kim H.J., Kim J.-K., Oh M.S., Kim S.G., Yu K.-H., Lee B.-C. (2015). A Low Baseline Glomerular Filtration Rate Predicts Poor Clinical Outcome at 3 Months after Acute Ischemic Stroke. J. Clin. Neurol..

